# Inborn errors of immunity at school: How can we do better?

**DOI:** 10.70962/jhi.20250120

**Published:** 2026-02-17

**Authors:** Laura Lopez-Seguer, Sonia Rodriguez-Tello, Miriam Gonzalez-Amores, Andrea Martin-Nalda, Stephany Zelada, Saida Ridao Manonellas, Sonia Galindo-Maycas, Carlota Villar, Jacques G. Riviere, Pere Soler-Palacin

**Affiliations:** 1 Infection and Immunity in Pediatric Patients Research Group. Vall d’Hebron Research Institute, Vall d’Hebron Barcelona Hospital Campus, Barcelona, Spain; 2 Barcelona Jeffrey Modell Diagnostic and Research Center for Primary Immunodeficiencies, Barcelona, Spain; 3 Barcelona PID Foundation, Barcelona, Spain; 4 Pediatric Infectious Diseases and Immunodeficiencies Unit, Children’s Hospital, Vall d’Hebron Barcelona Hospital Campus, Barcelona, Spain; 5 Universitat Autonoma de Barcelona, Barcelona, Spain

## Abstract

Adolescents with inborn errors of immunity (IEI) face physical limitations, social difficulties, psychological issues, and school absenteeism. Improving their school integration is essential. 13 adolescents with confirmed IEI attending high school, along with their parents, classmates, and teachers, participated in the study to evaluate social and academic adaptation indicators. A questionnaire was developed to gather perceptions on diagnosis, adaptation, understanding, socialization, coordination, and challenges. 13 adolescents (median age 13.8, IQR 13–15), 21 parents, 13 teachers, and 74 classmates participated. The illness impacted education, making it difficult for 50% of the students to keep up. 11 tutor teachers lacked specific knowledge and demanded more training. Most schools, 7/13, lacked absenteeism protocols, and 8/13 teachers were unaware of patient associations. Most classmates did not observe discriminatory behavior. IEI affect adolescents’ education. There is a need for better information, protocols to prevent absenteeism, improved coordination with healthcare teams, and contact with patient associations to enhance school adaptation.

## Teaser

Adolescents with inborn errors of immunity face school challenges across adaptation, understanding, coordination, and socialization. In a multi-informant survey (patients, parents, teachers, and classmates), we found a lack of absenteeism protocols, educator knowledge gaps, and limited school-healthcare coordination, despite positive peer acceptance. We propose co-developing standardized protocols with authorities and patient groups.

## Introduction

Primary immunodeficiencies (PIDs), also known as inborn errors of immunity (IEI), comprise more than 550 diseases of genetic origin that lead to an increased risk of severe and/or recurrent infections, autoimmunity, inflammation, allergic reactions, and neoplasms (1, 2).

IEI-related complications usually lead to recurrent hospital admissions and visits that increase the likelihood of restricted social contact, making these patients more susceptible to negative self-image, as well as a reduced perception of their own health-related quality of life and increased general fatigue (3, 4). Thus, a multidisciplinary approach to IEI patients and their families that includes psychological support is essential to improve patients’ quality of life, reducing the occurrence of general adaptation problems and the risk of suffering signs of anguish and depression (3, 5). In this sense, the International Patient Organization for Primary Immunodeficiencies (IPOPI) drafted a booklet that discusses the psychological effects that IEI can have on patients, parents, carers, and families and suggests ways to address them (6). In a broader view, rare disease impacts >300 million people globally, with 70% of these conditions starting in childhood (7). Some individuals living with a rare disease have physical or mental disabilities, which may have a greater impact on their health, and face various barriers that may hinder their full and effective participation in society on an equal basis with others. To that matter, the World Health Organization urges that those chronic conditions can increase school absenteeism and require individualized adaptations in educational (8, 9).

The educational community is a space for socializing and learning that contributes to normalizing the lives of children with chronic disease and enables them to define their personal and group identity. Intermittent absenteeism resulting from hospitalizations and long-term treatments is common in IEI students. This prompts the need to offer personalized counselling (10). All stakeholders in the field should aim to enable access to an equal educational opportunity and nondiscriminatory education for these children and adolescents by applying the necessary measures to face different educational stages (11). This need stands out especially as students with IEI become adolescents, considering its negative impact on social spheres (4). It is well known that the lack of knowledge on rare diseases and their consequences often limits social interaction with peers, as rejection or isolation leads to socio-affective problems and decreased academic performance (12, 13). However, implementing an inclusive approach in the schooling of teenagers with chronic diseases is challenging and, to date, in most countries, the welfare-state model does not resolve the majority of issues of children with IEI and their families (14).

Appropriate communication and coordination between healthcare professionals and schools is extremely important, with patients’ families being a key link between them (15, 16). However, due to the fear of discrimination, some family members may be reticent in providing information about the disease to their school (12, 15). Moreover, students themselves report difficulties associated with open communication, based on exclusion from school life, teachers’ reactions, and relationships with peers and friends (17, 18). Thus, communication and coordination strategies need reinforcing, as does the knowledge of health conditions through interprofessional education programs (11, 12).

The Barcelona PID Foundation is a nonprofit organization that was founded in 2014 at the initiative of a group of professionals dedicated to the care of IEI patients and their families. Its “I have a PID. I’m not alone.” program started in 2015 to provide psychological support and comprehensive care to children and adolescents with IEI. It is carried out in collaboration with a pediatric reference unit for IEI care in Catalonia, Northern Spain, that currently performs follow-up for more than 500 children and adolescents with a confirmed IEI diagnosis. As part of the program, the “PID at schools” project was created in 2016 with the aim to optimize the integration of children and adolescents with IEI at school and to conduct research activities to detect areas for improvement. Pediatric immunologists, researchers, nurses, and psychologists carry out specific coordination activities at patients’ schools with the active participation of patients, their teachers, and classmates.

In this setting, we conducted an observational study to evaluate the indicators of social and academic adaptation as reported by adolescents with IEI, their main caregivers, their classmates, and teachers.

## Results

20 adolescents with IEI aged between 12 and 18, who attended high school and were involved in the “I have a PID. I’m not alone.” program, were invited to participate. 13 of them—from 13 different schools—accepted (8 males; median age 13.8 [interquartile range [IQR]: 13–15]). Additionally, 21 parents (mothers 12/21), 13 tutor teachers, and 74 classmates (5.7 per patient) agreed to participate (see Table 1 for detailed demographic and clinical data). Reasons for not participating varied, with mistrust regarding confidentiality being the most common. Nine out of 13 participating schools were previously involved in the PID at schools project, with at least one coordination activity carried out between the school and the medical team.

**Table 1.** Main characteristics of PID patients and schools

​		
**PID patients (** ***N*** ** = 13)**		
Age	Median/IQR	13.8/13–15
Sex	M/F	8/5
Level of functioning	Level 0/1/2/3	4/3/4/2
Treatment	IgRT/SCT	6/1
PID Dx	X-linked agammaglobulinemia	3
	Ataxia telangiectasia	2
	Chronic granulomatous disease	2
	STAT3-LOF hyper IgE syndrome	1
	Autoimmune lymphoproliferative syndrome	1
	SAVI	1
	Combined immunodeficiencies with associated or syndromic features	1
	Autoinflammatory disorders (non-inflammasome-related conditions)	1
	Predominantly antibody deficiencies (severe reduction in at least two serum immunoglobulin isotypes with a normal or low number of B cells, CVID phenotype)	1
Frequency of psychology visits	Weekly/monthly/quarterly	2/7/4
Parents (*N* = 21)	Father/mother	9/12
**Schools (** ***N*** ** = 13)**		
Type of school	Public/private	11/2
Prior school coordination	Yes/no	9/4
Schools with classmate participation	Yes/no	9/4
Classmates (*N* = 74)	Male/female/non-binary	34/39/1

To calculate the patient’s level of functioning, we used the Eastern Cooperative Oncology Group performance status scale (19). CVID, Common variable immunodeficiency; Dx, diagnosis; IgRT, immunoglobulin replacement therapy; SAVI, STING-associated vasculopathy with onset in infancy; SCT, stem cell transplantation.

**Figure 1.**
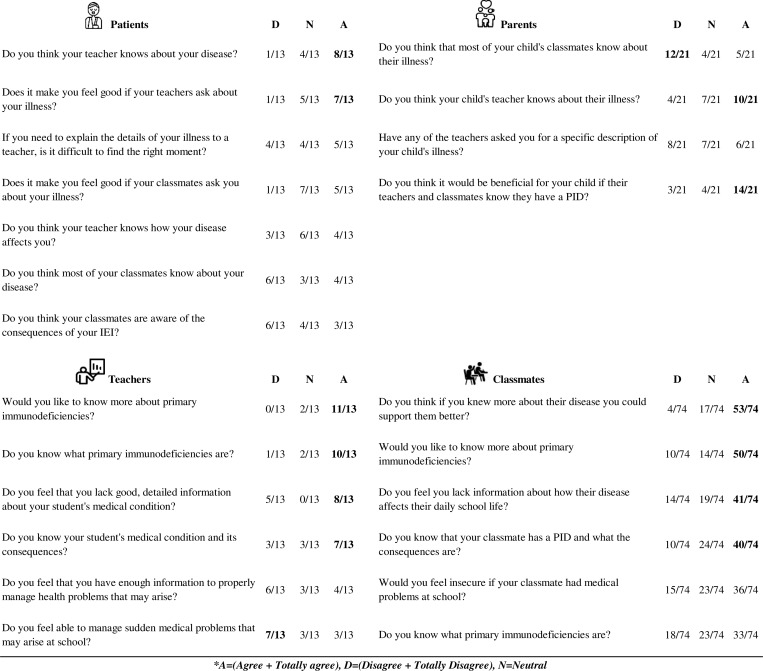
**Diagnostic results.** Top left: patient responses; top right: parent responses; bottom left: tutor teachers; bottom right: peer responses. Answers with agreement exceeding half of the participants are highlighted in bold.

Regarding diagnostic results, 8/13 patients and 10/21 parents felt that their tutor teachers knew about the patient’s disease, 7/13 patients liked to be asked by their teachers about their illness, and 14/21 parents thought that it would be beneficial for their child if their tutor teachers and classmates knew they had an IEI. However, 12/21 answered that their child’s classmates did not know about their child’s illness. Regarding tutor teachers, 11/13 would be interested to learn more about IEI, and 8/13 stated that they lacked detailed information about the medical conditions of the student in question. Finally, 40/74 of classmates stated that they knew that their classmate had an IEI and were aware of the consequences, but 41 of them felt they lacked information about the specific repercussions on the daily school life of the student in question. As occurred with tutor teachers, 50 of them would have been interested in receiving more information about IEI, and 53 believed that this would help them to provide optimal support to their affected classmates (see Fig. 1 for detailed answers).

In this area, we would like to highlight a comment that appeared in the free-text field of the questionnaires: “At times, being able to detect anxiety processes in our child has been complicated. These states of nervousness have been caused by returning to school after a long period of hospitalization and wanting to be at the same level as the rest of classmates” (parent).

**Figure 2.**
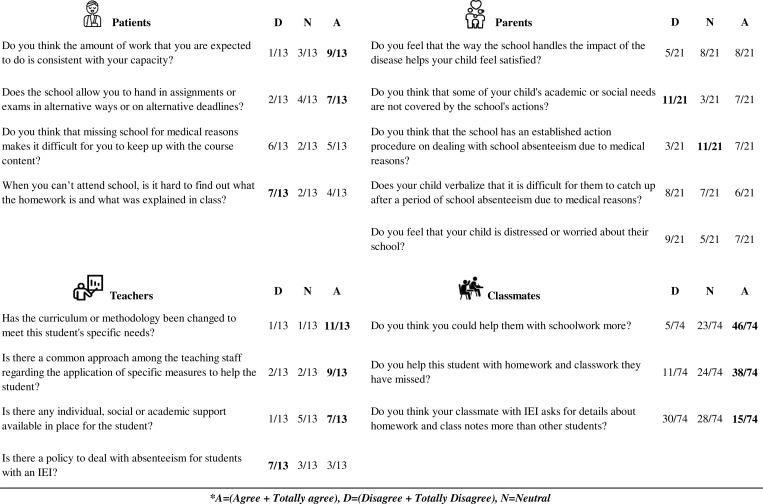
**Adaptation results.** Top left: patient responses; top right: parent responses; bottom left: tutor teachers; bottom right: peer responses. Answers with agreement exceeding half of the participants are highlighted in bold.

Regarding adaptation, 7/13 students with IEI said that they were allowed to hand in assignments or exams on a different day or via a different route. A total of 9/13 students felt that the amount of work that they are expected to do is in line with their capacity, but 11/21 parents gave a neutral score regarding whether the school had an established action procedure on dealing with school absenteeism due to medical reasons, and 11/21 reported that there are academic or social needs not covered by school actions. Moreover, teachers reported that 7/13 schools lacked a specific policy of action for students’ absenteeism, but 11/13 reported they had changed the curriculum or methodology to meet the students’ specific needs. Regarding classmates, 38/74 said they helped students with IEI with missed homework and classwork, but 46 felt they could do more. Only 15/74 responded that IEI students asked for more details about homework and class notes than other students (see Fig. 2 for detailed answers).

In this area, we would like to highlight these two comments from the free-text field of the questionnaires: “I think that knowing the diagnosis would allow me to better help my friend” (classmate); “I think that information about the disease at school is very important to help our child feel more supported and confident” (parent).

**Figure 3.**
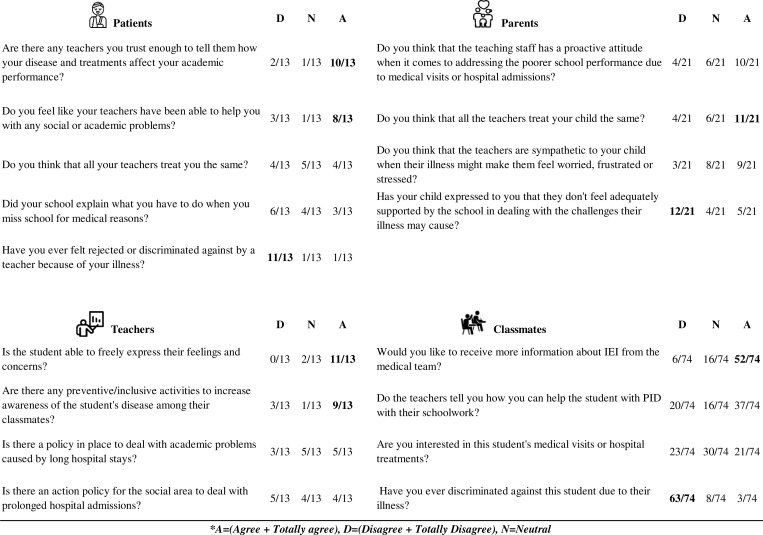
**Comprehension results.** Top left: patient responses; top right: parent responses; bottom left: tutor teachers; bottom right: peer responses. Answers with agreement exceeding half of the participants are highlighted in bold.

Regarding comprehension, 10/13 patients answered that there are tutor teachers they trust enough to tell them how their disease and treatments affect their academic performance, and 8/13 felt like their tutor teachers have been able to help them with social or academic problems. A total of 12/21 parents said that their children feel adequately supported by the school in dealing with the challenges their illness may cause. Moreover, 9/13 tutor teachers said that there are preventive/inclusive activities to increase awareness of the students’ disease among their classmates, and 11/13 believed that IEI students are able to freely express their feelings and concerns. Furthermore, 52/74 classmates expressed a willingness to receive more information on IEI from the medical team, and 63 reported not having any discriminatory behavior towards their classmate with IEI due to their illness (Fig. 3).

In this area, we would like to highlight these two comments: “Having a protocol would be very helpful to train teachers how to act properly, since, despite their willingness, what to do is not clear” (parent); “I think that I already have enough information to be able to help the student, but any additional educational activity would be highly welcomed” (tutor teacher).

**Figure 4.**
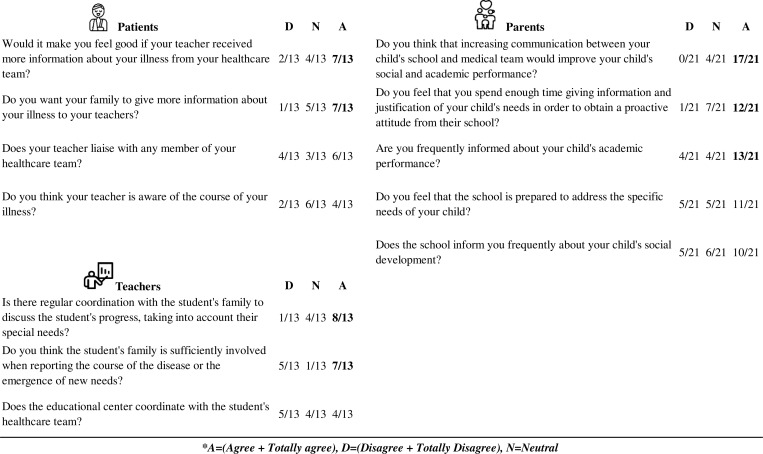
**Coordination results.** Top left: patient responses; Top right: parent responses; bottom left: tutor teachers. Answers with agreement exceeding half of the participants are highlighted in bold.

When examining coordination, results revealed that 7/13 patients with IEI expressed a desire for improved coordination between their tutor teachers and healthcare team, including regular updates on their health status, and 7/13 wanted their family to give more information about their illness to their teachers. A total of 17/21 parents also felt that increasing communication between the school and the medical team could improve their child’s social and academic performance. In that regard, 7/13 teachers felt that parents were actively and sufficiently involved when reporting on the course of their child’s disease or the emergence of new needs, and 8/13 answered that they coordinated with the family on a regular basis to closely monitor the student’s status to discuss their progress, taking into account their special needs. A total of 13/21 parents felt that they were frequently informed about their child’s academic performance (Fig. 4).

The comment “It would be beneficial to regularly coordinate with the clinical unit in order to adjust universal, additional and intensive measures at school” (tutor teacher) is of relevance in this section.

**Figure 5.**
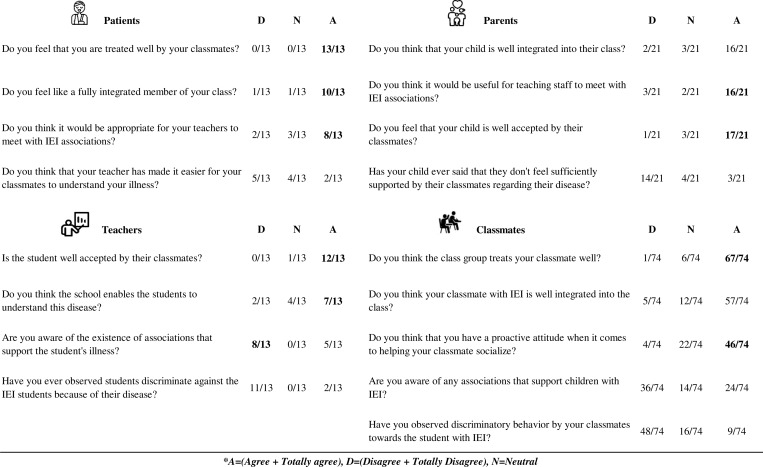
**Socialization results.** Top left: patient responses; top right: parent responses; bottom left: tutor teachers; bottom right: peer responses. Answers with agreement exceeding half of the participants are highlighted in bold.

Finally, when analyzing socialization, all 13 patients reported that they felt treated properly by their classmates, and 10/13 felt fully integrated. This was also expressed by 67/74 and 57/74 of their classmates, respectively. A total of 12/13 tutor teachers and 17/21 parents mentioned that students with IEI were well accepted by their classmates. Moreover, 46/74 classmates thought that they showed a proactive attitude when it came to helping their classmates socialize, and 48/74 did not observe any discriminatory behavior from other students towards their peers with an IEI. Regarding patient associations, 8/13 tutor teachers did not know that IEI foundations or associations existed, but 16/21 parents and 8/13 patients thought that it would be useful for teaching staff to meet with IEI patient associations (Fig. 5).

**Figure 6.**
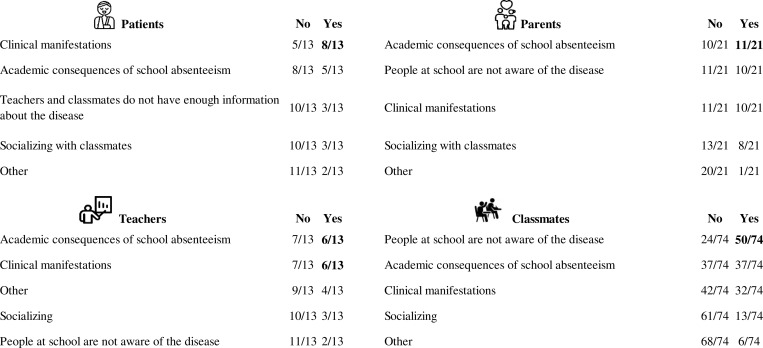
**Challenging areas results.** Top left: patient responses; top right: parent responses; bottom left: tutor teachers; bottom right: peer responses. Answers with agreement exceeding half of the participants are highlighted in bold.

Upon examining the most challenging areas to manage, 8/13 patients emphasized having active clinical manifestations of the disease as the most challenging situation, while 11/21 parents reported that the main challenge was the consequences of school absenteeism due to the disease. These two areas were also chosen by 6/13 tutor teachers. Finally, 50/74 classmates reported that the most difficult area to manage when helping a patient with IEI is their lack of knowledge about the disease (see Fig. 6 for detailed answers).

## Discussion

This study shows IEI’s impact on adolescents’ schooling at different levels and that there is a need to provide recommendations for home, school, community, and healthcare settings to improve the quality of life of individuals with rare diseases during childhood and adolescence. Disease complications, frequent hospitalization, and medical visits contribute to school absenteeism and social isolation, as shown by Kuburovic et al. (3). This is particularly true in emotional, social, and school psychosocial domains, as these children are at an increased risk of experiencing severe anxiety and depressive symptoms. The current study adds to the evidence in the literature that, while students and families report positive social acceptance in schools, the educational and healthcare system lacks preparedness to meet their needs effectively.

School absenteeism due to disease complications significantly impacts quality of life and emotional well-being due to restricting social contact. Patients are thus more susceptible to experiencing negative self-perception, decreased perception of their own health, and an increase in general fatigue, as previously demonstrated by our group (4). The study by Xiao et al. represents a literature review focusing on health-related quality of life (HRQoL) in IEI patients. It stresses the need for more extensive and systematic research in this area, emphasizing the importance of a multidisciplinary approach. Despite advances in medical treatments for IEI, there is a crucial need to focus on HRQoL to improve patient satisfaction and overall well-being (20). Our findings reveal a lack of structured and systematic protocols within schools to address school absenteeism and its consequences and emphasize the need for early biopsychosocial assessment and support to prevent long-term mental health consequences. Digital or hybrid education might help minimize absenteeism in chronic diseases (21). However, when implementing them, it is crucial to manage the possible increase of inequities and digital breaches to avoid creating new barriers (21).

In addition to specific protocols dealing with school absenteeism, our study emphasizes gaps in disease-related knowledge among educators and peers. Our data show that, despite more than half of classmates being aware of the existence of the disease, the vast majority reported lacking specific knowledge about the disease and its repercussions on daily school life. Lack of knowledge is described by almost every stakeholder as a limiting factor. As observed by Hinton et al., teachers often lack formal training in chronic condition management, and this may limit their ability to address the needs of students with IEI (22). Even when aware of the disease, truly understanding the academic consequences and implications is inadequate. As expressed by García-Perales et al., there is an urgent need for widespread educator training to foster true inclusive education, using knowledge and understanding of the characteristics and potential of students with rare diseases (23). Across contexts, teachers commonly face limited condition-specific training, workload, resource constraints, and the absence of a designated school-health liaison, which hinder implementation of inclusive practices (9, 22, 24). We also acknowledge that policies and accommodations vary internationally; settings with nationwide school-health frameworks may face different barriers, whereas our findings reflect the Spanish/Catalan context, where condition-specific guidance for rare diseases remains fragmented. A brochure from the IPOPI tries to fill this gap by offering guidance on how schools can best support these students from a health point of view but also addressing education, absenteeism, and bullying, among other issues. In Spain, several associations and foundations dedicated to various rare diseases are actively advocating for better support within schools and providing guidance tailored to specific conditions. We believe in the importance of merging forces from the different stakeholders to carry out common protocols in the future, using expertise networks for dissemination, such as European Reference Networks (ERNs), the corresponding scientific societies, as well as patient associations or foundations (19).

Coordination between healthcare teams, schools, and families is also a recurrent unmet need highlighted by our study. Although essential, it is usually insufficiently implemented, as shown by Verger et al. and Rosselló et al., who stress the importance of interdisciplinary approaches to meet the needs of children with rare diseases (15, 24). Our findings support these observations, highlighting the need for regularly updated structured communication pathways. Moreover, coordination should explicitly extend to healthcare transition from pediatric to adult services and its interface with postsecondary education/employment supports; although consensus recommendations exist in IEI (e.g., ERN for Rare Immunodeficiency, Autoinflammatory and Autoimmune Diseases), empirical data linking transition processes to education or work outcomes are currently lacking (19). At our center, a hospital-wide transition program is in place; future work will formalize links with schools/universities and vocational services to close this gap.

Patient associations play a pivotal role in raising awareness, providing resources, and advocating inclusive education. As Gaintza et al. demonstrate, these organizations enhance community understanding of rare diseases and promote positive attitudes within school communities (25). Paz-Lourido et al. also show a high acceptance of educational agents for the school environment in rare disease both in primary and secondary schools (26). Our study aligns with these findings, showing strong support from educators, classmates, and families for the involvement of patient associations to foster adaptive measures and prevent discrimination. Empowering children and adolescents with IEI by following a specific and well-structured program improves their QoL, as shown by Fasshauer et al. (27). Despite the lack of specific protocols and knowledge, one distinguishing trait of our findings is that our data suggest that social acceptance among peers is generally positive, in contrast to other studies like that by Adama et al., where stigma and bullying were highly prevalent among patients with rare diseases (28). This difference might be explained by one of the main limitations of our study: the relatively small sample size of schools and individuals with an IEI, though comparable to that of similar studies, and also by the differences regarding the rare diseases studied for the 41 families in Adama’s studies. Additionally, the inclusion of participants already enrolled in psychological support programs like PID at schools may have created a selection bias, potentially underestimating the challenges faced by others without such resources. Given the heterogeneity of rare diseases and even within IEI, differences across studies are expected. Likely determinants include: IEI groups, Eastern Cooperative Oncology Group performance status, sex/gender, socioeconomic context, school settings (public vs. private; urban vs. rural), prior exposure to support programs, and parental engagement. Many of these variables are inconsistently reported in the literature, which limits comparability and interpretation. In our cohort, the sample size precluded adjusted or stratified analyses by these dimensions. Exploratory stratifications by school sector and parent sex were performed but not presented in our study because group sizes were extremely imbalanced and underpowered, yielding no consistent trends. By May 2021, restrictive measures related to the COVID-19 pandemic had already been considerably relaxed in our school settings, and students attended classes wearing ear-loop face masks, generally with a greater sense of normalcy compared to the most acute moments of the pandemic. However, the health situation could have influenced perceptions of special attention toward children with immunodeficiencies, which constitutes another possible bias. Lastly, the cross-sectional design limits the evaluation of long-term outcomes and the impact of interventions over time, highlighting the need for larger, multicenter, longitudinal studies. With those limitations in mind, in the following years, our group is aiming to apply a broader, multicenter, validated evaluation, including the use of the RE-AIM framework, to enable greater coverage to overcome most of these limitations (29). Also, a stepwise validation program including psychometric modeling has been planned with the goal of producing a robust, adoptable instrument for IEI school settings more scalable and comparable to other settings.

### Conclusion

Adolescents with IEIs face significant challenges impacting their education, emotional well-being, and overall QoL. This study highlights critical gaps and unmet needs in educational and healthcare systems to support disease impact in the school environment. Specifically, the absence of structured protocols to address absenteeism, limited disease-related knowledge among educators, and insufficient coordination between families, schools, and healthcare teams continue to be significant barriers. Patient associations and foundations already advocate for stronger school support and condition-specific guidance. Building on these efforts, we propose co-developing standardized protocols and resources with education authorities and disseminated them through expert networks—such as ERNs, relevant scientific societies, and patient associations. Co-development should include all stakeholders. Our next steps are to develop, pilot, and evaluate these materials with stakeholders to enable scalable, system-level adoption.

Despite these challenges, the findings highlight positive social acceptance from peers and the important role of patient associations in promoting awareness and fostering inclusivity. Addressing the identified gaps through interdisciplinary collaboration, educator training, and the development of structured action plans is essential. Future multicenter studies with larger samples are needed to confirm these findings and guide the implementation of effective, inclusive educational strategies to improve the quality of life for adolescents with an IEI.

## Materials and methods

### Study design

A 2-mo, prospective, cross-sectional, observational study was initiated in May 2021. At that time, children in Catalonia attended school with the sole restriction of wearing an ear-loop face mask to class.

### Questionnaire design and measures

Four different questionnaires (one for each category: patients, parents, tutor teachers [teachers who are primarily responsible for the students in a specific class], and classmates) were co-created by a multidisciplinary team, including pediatric immunologists, pediatric psychologists, a specialist nurse practitioner together with the advisory patient council of the Barcelona PID Foundation, composed of 12 adolescents and adults with IEI. This co-design provided the basis for initial face and content validity and ensured that items were context-specific and age/role appropriate. Because the aim of this study was an exploratory mapping across informants, items were tailored to each respondent group rather than forced to be parallel. The questionnaire comprised five evaluative specific areas (knowledge of the IEI diagnosis, perception of patient’s adaptation at school, the school’s understanding of the disease’s impact, perception of patient–family–school coordination, and perception of patients’ socialization) and one final area that asked for a selection of the two most challenging disease-related areas to sustain individually. A Likert rating scale was used to evaluate answers. Furthermore, a free-text comments section was available to add reflections if needed for all questions and areas.

Results with consensus of more than half of the participants were considered relevant, included in the Results section of this manuscript, and bolded in Fig. 1, 2, 3, 4, 5, and 6.

### Data collection

Data were collected at one time point during the study period. Patients, parents, and tutor teachers were invited to participate by telephone or email, whereas classmates were invited by their own tutor teachers.

The questionnaires were sent to all participants via email through a link to the REDCap platform (Vanderbilt University). Each patient, at least one parent, the head teacher, and all classmates willing to participate answered the questionnaires.

### Statistical analysis

Qualitative variables were described as numbers and percentages. Quantitative variables were described as mean and standard deviation or median and IQR. Confidence intervals for all analyses were considered at 95%. All statistical analyses were performed with R software, Version 4.2.0 (2022-04-22 ucrt), The R Foundation for Statistical Computing. This study was not powered for psychometric modeling; no factor or test–retest analyses were performed in the present pilot, which focuses on descriptive results across informants.

## Ethics

All the information collected during the study was strictly confidential. Participants signed the study informed consent (IC); participants ≥12 and <18 years signed the informed assent along with their legal guardian. The study followed the World Medical Association’s Declaration of Helsinki and obtained ethical approval from Vall d’Hebron Barcelona Hospital Campus Research Ethics Committee (PR(AMI)192/2021). IC was obtained from all participants.

## Data Availability

All data generated or analyzed during this study are included in this published article. If needed, detailed data are available from the corresponding author upon reasonable request.
